# Lipocalin‐2 induced LDHA expression promotes vascular remodelling in pulmonary hypertension

**DOI:** 10.1111/cpr.13717

**Published:** 2024-07-18

**Authors:** Guoliang Wang, Shenghua Liu, Xiaohui Kong, Hong Jiao, Feng Tong, Zhangke Guo, Meng Zhang, Xiaoxing Guan, Na Ren, Wanzhen Li, Lihua Qi, Yingjie Wei

**Affiliations:** ^1^ Department of Tumor and Immunology, Beijing Pediatric Research Institute, Beijing Children's Hospital Capital Medical University, National Center for Children's Health Beijing China; ^2^ State Key Laboratory of Cardiovascular Disease, Fuwai Hospital, National Center for Cardiovascular Disease Chinese Academy of Medical Sciences and Peking Union Medical College Beijing China; ^3^ Department of Cardiac Surgery, Beijing Children's Hospital Capital Medical University, National Center for Children's Health Beijing China; ^4^ Department of Pathology, Beijing Children's Hospital, Capital Medical University National Center for Children's Health Beijing China; ^5^ Department of Clinical Laboratory Center, Beijing Children's Hospital Capital Medical University Beijing China; ^6^ Department of Lipidomics Experimental Platform, State Key Laboratory of Molecular Developmental Biology, Institute of Genetics and Developmental Biology Chinese Academy of Sciences Beijing China; ^7^ Department of Human Anatomy, Histology and Embryology, School of Basic Medical Sciences Peking University Health Science Center Beijing China

## Abstract

Aerobic glycolysis is involved in the pathogenesis of pulmonary hypertension (PH). The mechanisms by which glycolysis is increased and how it contributes to pulmonary vascular remodelling are not yet fully understood. In this study, we demonstrated that elevated lipocalin‐2 (LCN2) in PH significantly enhances aerobic glycolysis in human pulmonary artery smooth muscle cells (PASMCs) by up‐regulating LDHA expression. Knockout of *Lcn2* or having heterozygous LDHA deficiency in mice significantly inhibits the progression of hypoxic PH. Our study reveals that LCN2 stimulates LDHA expression by activating Akt‐HIF‐1α signalling pathway. Inhibition of Akt or HIF‐1α reduces LDHA expression and proliferation of PASMCs. Both Akt and HIF‐1α play critical roles in the development of PH and are suppressed in the pulmonary vessels of hypoxic PH mice lacking LCN2. These findings shed light on the LCN2‐Akt‐HIF1α‐LDHA axis in aerobic glycolysis in PH.

## Graphical abstract

- Knockout of lipocalin‐2 (LCN2) suppresses Akt phosphorylation, HIF‐1α and LDHA expression, and pulmonary hypertension (PH) progression.
- Heterozygous LDHA deficiency suppresses PH progression.
- LCN2 promotes LDHA expression by activating Akt‐HIF‐1α signalling axis.
- LCN2‐Akt‐HIF‐1α‐LDHA axis is a potential target for PH treatment.
- Up‐regulated LCN2 promotes LDHA expression and aerobic glycolysis via activation of Akt‐HIF‐1α signalling, facilitating the metabolic abnormalities and pulmonary artery smooth cell proliferation in pulmonary hypertension.

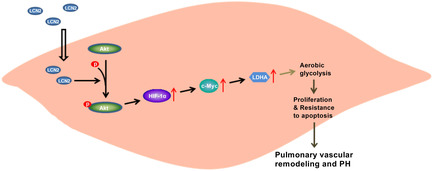

## 1 INTRODUCTION

There is abundant evidence indicating that pulmonary hypertension (PH) is associated with metabolic abnormalities.^1^, ^2^ Studies have shown higher levels of glycolysis and reduced glucose oxidation in pulmonary vascular cells of both animal models and PH patients,^3^, ^4^, ^5^, ^6^ resembling the Warburg effect observed in rapidly dividing tumour cells.^7^, ^8^ The close link between metabolic changes and the phenotype of pulmonary vascular cells underpins the metabolic theory of PH, that increased proliferation and inflammatory activation may occur when cellular metabolism switches to aerobic glycolysis.^1^, ^4^ However, the precise mechanisms driving cell metabolic reprogramming in PH remain largely unknown.

Lactate dehydrogenase (LDH) is an enzyme composed of either LDHA or LDHB subunits that combine to form a tetramer, facilitating the reversible conversion of pyruvate to lactate.^9^ In cancer cells, dysregulated LDHA catalyses the conversion of pyruvate to lactate, concurrently regenerating NADH to NAD^+^.^7^, ^10^ This diversion of metabolic precursors of pyruvate into the pentose phosphate pathway contributes to the growth of cancer cell by supplying metabolic building blocks.^7^ Furthermore, the heightened levels of lactate resulting in a lower pH environment can enhance tumour angiogenesis and aid in immune escape.^11^, ^12^ Up‐regulated LDHA has been associated with critical functions in cell proliferation and apoptosis in different types of cancers.^13^, ^14^, ^15^ Inhibiting LDHA has been shown to slow down tumour growth and reduce the aggressiveness of tumour cells,^16^, ^17^ highlighting the critical role of LDHA in tumour progression. However, the potential role of LDHA in regulating glucose metabolism in PH has not been investigated.

Lipocalin‐2 (LCN2) is a 25‐KDa protein known for its involvement in iron transport and innate immunity.^18^ Recent reports have linked LCN2 to multiple pathophysiological processes, including glucose metabolism, apoptosis and insulin resistance.^19^, ^20^, ^21^, ^22^ Elevated expression of LCN2 has been observed in various types of cancer and is implicated in different aspects of tumour progression.^22^ Both a recent report^23^ and our previous studies^24^, ^25^, ^26^ have highlighted the up‐regulation of LCN2 in PH, suggesting a potential role in promoting the proliferation of pulmonary artery smooth muscle cells (PASMCs). However, the exact contribution of LCN2 to PH pathogenesis and the underlying mechanisms require further investigation. Recent studies have also highlighted the metabolic regulatory role of LCN2 in cardiovascular conditions,^27^, ^28^ particularly in glucose metabolism.^19^, ^28^ Based on these findings, we propose that LCN2 may play a role in driving the metabolic shift towards glycolysis in PH.

The aim of this study is to explore the role of LCN2 in driving the shift towards glycolysis in the context of PH. In this work, we have identified a novel molecular pathway in which elevated levels of LCN2 enhances the expression of LDHA by activating Akt and hypoxia inducible factor‐1 alpha (HIF‐1α) signalling. This leads to a switch from glucose metabolism to aerobic glycolysis, ultimately fostering the proliferation of PASMCs while reducing apoptosis in PH.

## 2 MATERIALS AND METHODS

### 2.1 Study design

The study design was based on our previous reports^24^, ^25^, ^26^ that LCN2 is up‐regulated in PH and can stimulate PASMC proliferation in vitro. To elucidate the pathological role of LCN2 in PH, we employed a hypoxic PH model utilizing *Lcn2* KO mice. These mice exhibited significant improvement in haemodynamics and histology compared to hypoxic control mice, providing evidence of the detrimental impact of LCN2 in PH. Then, using metabolomics methods, we found a significant decrease in glycolytic metabolites in the lung tissue of *Lcn2* KO mice compared to controls. Further investigation into key glycolysis enzymes unveiled a significant decrease in LDHA expression in *Lcn2* KO mice. In contrast, LDHA expression was notably elevated in PH patients and classic rat PH models. Using the hypoxic PH model, we determined that even partial LDHA deficiency led to substantial improvement in the pathophysiology of PH, indicating the involvement of LCN2‐induced LDHA expression in driving PH‐related changes. Additionally, experiments conducted on cultured human PASMCs demonstrated that suppressing LDHA expression resulted in reduced cellular glycolysis, inhibiting cell proliferation and promoting apoptosis. Through pharmacological interventions, we identified the pAkt‐HIF‐1α signalling pathway through which LCN2 enhances LDHA expression in PH. This signalling mechanism was corroborated by molecular alterations observed in mouse and rat PH models.

### 2.2 Experimental animals

All animal protocols and surgical procedures were approved by the local animal care committee and were performed in accordance with the ethical standards outlined in the 1964 Declaration of Helsinki and its subsequent amendments. Mouse strains possessing LCN2 knockout (C57BL/6J‐*Lcn2* ^em1GemP^; C57BL/6JGpt, *Lcn2* KO), LDHA knockout (C57BL/6J‐*Ldha* ^em1GemP^, *Ldha* KO), as well as their littermate control mice were purchased from Gempharmatech (Nanjing, CHN). Sprague‐Dawley rats were purchased from Charles River Laboratories (Beijing, CHN).

### 2.3 Hypoxic PH model

*Lcn2* KO mice, *Ldha* KO mice and their littermate control counterparts (age 10–12 weeks, half male and half female) were exposed to room air (normoxia) or 10% oxygen (hypoxia) within a transparent plastic chamber (35 × 45 × 25 cm) equipped with ventilation openings. The oxygen concentration (10%) was maintained using a Proox Oxygen Controller (BioSpherix, Lacona, NY), while a quiet fan ensured efficient gas mixing within the chamber. Gas renewal occurred at a rate of 1–2 L/min to uphold low levels of CO_2_ and NH_3_ within the chamber. Relative humidity within the chamber was maintained below 50%. The mice had ad libitum access to standard mouse chow and drinking water. Subsequent to a 4‐week exposure period, evaluations of haemodynamics and pulmonary vascular remodelling were performed.

### 2.4 Monocrotaline‐induced PH rat model

Male Sprague‐Dawley rats (aged 5 to 6 weeks; weighing 200 to 250 g) were administered with single subcutaneous injection of 60 mg/kg monocrotaline (MCT) or saline. After 3 weeks, echocardiography and histological analyses were conducted to evaluate pulmonary hypertension.

### 2.5 Sugen/hypoxia pulmonary hypertension rat model

Male Sprague‐Dawley rats (aged 5 to 6 weeks; weighing 200 to 250 g) were subcutaneously injected with SU5416 (20 mg/kg) and exposed to 10% oxygen (hypoxia) for 3 weeks. The oxygen concentration was controlled using a Proox Oxygen Controller. Control rats, matched in terms of sex, age and weight, were kept under normoxia for 3 weeks. All rats had unrestricted access to standard rat chow and drinking water. After the 3‐week period, haemodynamic measurements and assessments of pulmonary vascular remodelling were conducted.

### 2.6 Echocardiography

Transthoracic echocardiography was performed using the Visual Sonics Vevo 3100 ultrasound machine with a 40 MHz ultrasound probe (MS400D). Briefly, mice and rats were anaesthetized with continuous isoflurane inhalation (1.5%–3.0%), and positioned in a supine orientation on a warm pad. The chest fur was shaved, and measurements such as right ventricular (RV) wall thickness during diastole (using M‐mode in the parasternal long axis view), pulmonary artery (PA) acceleration time (PAAT, from pulsed Doppler mode in a modified parasternal long axis view) and cardiac output (CO, from M‐mode in the parasternal short axis view) were taken.

### 2.7 Right ventricular pressure assessment by closed‐chest catheterization

Rats or mice were placed on a warm pad in a supine position after anaesthetized with isoflurane inhalation (1.5%–3.0%). A right neck incision was made, and the right jugular vein was gently exposed and separated. Subsequently, a 1.9‐F catheter connected to a pressure transducer (AD Instruments) was introduced into the RV through the right jugular vein. RV pressure was recorded continuously for 20–30 s, and the final RV pressure values were determined by averaging 5–8 consecutive cardiac cycles.

### 2.8 Histological analyses

Following the haemodynamic measurements, mice and rats were anaesthetized with ketamine hydrochloride (60 mg/kg, i.p.) and xylazine (8 mg/kg, i.p.), and euthanized through thoracotomy. The blood in the pulmonary circulation was flushed out by infusing phosphate buffered saline (PBS) cell through the pulmonary artery. Subsequently, the heart and lungs were removed. The RV free wall, left ventricle (LV) and septum were meticulously dissected and weighed individually to calculate the Fulton index (weight ratio of RV to LV + septum). The right lung was frozen in liquid nitrogen for further analysis, while the left lung was fixed in a 4% paraformaldehyde solution for 24 h and then embedded in paraffin. Slides of 5 μm thickness were stained with haematoxylin and eosin (H&E) for morphological examination. The Olympus BX‐53 digital camera and ImageJ software (http://rsbweb.nih.gov/ij/) were employed to analyse the slides. The total vascular area (bounded by adventitia) and lumen area (bounded by basement membrane) in 15–20 muscular arteries with a diameter of 50–100 μm per lung slide were outlined to assess pulmonary arterial wall thickness: wall thickness = (total vascular area − lumen area)/total vascular area.

### 2.9 Metabolomics analysis

Lung tissue samples were diced into small fragments. A 10 mg sample was measured and mixed with 20 μL of deionized water before being homogenized for 3 min. Following this, 120 μL of cold methanol (containing an internal standard) was added and homogenized once more. The samples were then centrifuged at 4°C, 18000 g for 15 min. Next, 20 μL of the supernatant was transferred into a 96‐well plate, and sequentially, 20 μL of 200 mM 3‐NPH and 20 μL of 120 mM EDC were added into each well. After a 60‐min incubation at 30°C, 1450 rpm, the samples were diluted with cold methanol and analysed by UPLC‐MS/MS (Acquity‐I Xevo TQ‐S). Each tested sample was a combination of two distinct lung samples from the same group.

### 2.10 Primary mouse pulmonary artery smooth muscle cells isolation

Primary mouse PASMCs were isolated through an enzymatic digestion procedure. Briefly, pulmonary artery branches were dissected from lung lobes under a stereomicroscope. After carefully removing the fibrous adventitia layer, the blood vessels were longitudinally opened, and the endothelial layer was scraped away. The remaining blood vessels were cut into approximately 1 mm segments. These segments were washed with cold PBS and then digested at 37°C for 60 min in Hank's balanced salt solution containing 0.1% collagenase. Subsequently, the cell suspensions were centrifuged at 100 g for 5 min, and PASMCs were resuspended and cultured in M199 medium (Gibco) supplemented with 10% fetal bovine serum. The purity and authenticity of the cells were confirmed through immunofluorescent staining against smooth muscle actin.

### 2.11 Cell culture

Primary human PASMCs were purchased from ScienCell (#3110) and cultivated in Smooth Muscle Cell Medium (#1101; ScienCell). The cells were maintained in a sterile, humidified incubator at 37°C with 5% CO_2_. After reaching 90%–95% confluence, the cells were subcultured using 0.25% trypsin‐EDTA. Cells from the seventh to ninth passages, acclimated in 0.5% fetal bovine serum and growth factor‐free SMCM for 24 h, were utilized in all experiments. For hypoxic treatment, preconditioned cells were subjected to a hypoxia chamber (0.1% O2, Billups‐Rothenberg), followed by LCN2 intervention (2 nM for 24 h) after 3 h of cell hypoxic exposure.

### 2.12 Immunofluorescent confocal microscopy

To determine the content of LDHA, p‐Akt, HIF‐1α, collagen‐I, Ki67 and TUNEL in pulmonary arterioles, double immunostaining of LDHA/α‐actin, p‐Akt/α‐actin, HIF‐1α/α‐actin, collagen‐I/α‐actin, Ki67/α‐actin and TUNEL/α‐actin was performed on lung tissue slides from PAH and control animals. Initially, the lung slides were incubated with a rabbit polyclonal antibody against LDHA (dilution 1:100), p‐Akt (dilution 1:100), HIF‐1α (dilution 1:100), collagen‐I (dilution 1:100), Ki67 (dilution 1:100) and a mouse monoclonal antibody against α‐actin (dilution 1:500) overnight. Subsequently, they were treated with goat anti‐rabbit IgG Alexa Fuor 488 and goat anti‐mouse IgG Alexa Fuor 594 (dilution 1:500). The slides were sealed with mounting solution containing anti‐fade reagent and examined using an OLYMPUS BX53 microscope. The yellow colour indicates co‐localization of LDHA, p‐Akt, HIF‐1α and collagen‐I with α‐actin. The area of yellow colour was quantified using ImageJ.

### 2.13 Western blotting analyses

Frozen lung tissue was homogenized in RIPA lysis buffer and centrifuged at 15000 g and 4°C for 10 min. Cells were trypsinized, washed twice with PBS and then lysed with RIPA lysis buffer on ice for at least 30 min. Subsequently, the lysates were centrifuged at 15000 g and 4°C for 10 min. Protein concentration was determined using the BCA Protein Assay Kit with BSA as the standard. Equal amount of protein (50 μg for rat lung samples, 20 μg for cell samples) from each sample was mixed with 5× sample loading buffer and boiled for 5 min. The protein mixtures were separated on a 12% SDS‐PAGE gel, transferred to a nitrocellulose membrane and probed with the following antibodies: rabbit anti‐Lcn2, rabbit anti‐LDHA, rabbit anti‐HK, rabbit anti‐PFK, rabbit anti‐PDK1, mouse anti‐pAkt, mouse anti‐tAkt, mouse anti‐HIF‐1α, mouse anti‐c‐Myc, mouse anti‐PKLR, mouse anti‐GAPDH, mouse anti‐β‐actin, goat anti‐rabbit IgG and goat anti‐mouse IgG. Protein bands were visualized using chemiluminescene and quantified by densitometry with ImageJ software.

### 2.14 Quantitative real‐time polymerase chain reaction (RT‐PCR) analyses

Total RNA was extracted from cells and frozen mouse lung tissues using Trizol Reagent (Invitrogen). The RNA was then reverse‐transcribed with Oligo (dT) and Transcriptor Reverse Transcriptase (Roche, GA) according to the manufacturer's instructions. Real‐time quantitative PCR was performed on an Applied Biosystems 7300 Fast Real‐Time PCR System (ABI, USA) using SYBR Green PCR Master Mix. The expression levels of gene transcripts in the test samples were normalized to the internal standard β‐actin. The sequences of gene‐specific primers are as follows: LDHA, 5′‐GTC CAG CGT AAC GTG AAC AT‐3′ and 5′‐CCA AGC CAC GTA GGT CAA GA‐3′; ACTB, 5′‐TGT TTG AGA CCT TCA ACA CC‐3′ and 5′‐GAG GGC ATA ACC CTC GTA G‐3′.

### 2.15 Lactate assay

Intracellular lactate levels were determined using a lactate assay kit (Sigma; MAK064) according to the manufacturer's instructions. Briefly, PASMCs were seeded in six‐well plates. After indicated treatments, cells were collected and lysed in RIPA lysis buffer. Subsequently, 50 μL of diluted samples were used to determine lactate concentrations as per the manufacturer's protocol. The data were read using a Multiscan GO reader (Thermo) and then normalized to protein content.

### 2.16 Lactate dehydrogenase (LDH) activity assay

LDH activity in PASMCs was determined according to the manufacturer's instructions (Sigma; MAK066). A total of 1 × 10^6^ cells were suspended in 500 μL LDH assay buffer and centrifuged at 10000 g and 4°C for 15 min. The LDH activity was measured at 450 nm using a Multiscan GO reader (Thermo).

### 2.17 RNA interference in PASMCs

The expressions of LDHA were silenced using siRNA technology. LDHA siRNA (5′‐GGGAGAAAGCCGUCUUAAUdTdT‐3′) and control siRNA were synthesized by Beiruisike (Beijing, CHN). The siRNAs were transfected into PASMCs with MaxFect Lipo3000 transfection reagent (GZ30003, GeneZe, CHN) according to the manufacturer's protocol. Three days after transfection, the cell medium was replaced with serum‐free medium for 24 h before the indicated treatments.

### 2.18 Extracellular flux analyses (Seahorse)

The Seahorse Extracellular Flux analyser (XFe96, Agilent) was used to evaluate glycolysis through measuring the extracellular acidification rate (ECAR) and mitochondrial respiration by measuring the oxygen consumption rate (OCR) of human PASMCs. The PASMCs were cultivated in XF96 microplates (1.5 × 10^5^ cells/well). Following the designated treatment, cells were rested in a non‐buffered assay medium in a non‐CO_2_ incubator for 60 min. Parameters associated with oxidative phosphorylation were evaluated using Seahorse XF Cell Mito Stress Test Kit, involving three injections: (1) 1 μM oligomycin; (2) 0.25 μM FCCP; and (3) 0.5 μM rotenone/antimycin A. Parameters linked to glycolysis were determined using the Seahorse XF Glycolysis Stress Test Kit with three injections: (1) 10 mM glucose; (2) 1 μM oligomycin; and (3) 50 mM 2‐DG.

### 2.19 In vitro cell proliferation and apoptosis measurements

Cells were fixed with 4% paraformaldehyde. Ki67 immunofluorescent staining was performed to evaluate cell proliferation, while the TUNEL assay was performed to assess cell apoptosis. The percentage of Ki67‐positive or TUNEL‐positive cells was calculated by dividing the number of positive cells by the total number of nuclei (DAPI). A minimum of 400 cells from 5 to 8 randomly selected fields were counted per group. All experiments were performed at least three times.

### 2.20 Statistical analyses

Each experiment was performed in triplicate and repeated at least three times. Differences between variables were assessed by Mann–Whitney *U* test or Kruskal–Wallis test, where appropriate. Dunn's test was utilized for pairwise comparisons if the difference among multiple groups was statistically significant in the Kruskal–Wallis test. Data were shown as mean ± SEM for ‘*n*’ experiments. A bilateral *p* value of less than 0.05 was considered statistically significant. Statistical analyses were conducted using GraphPad Prism 8.0. Analyses of online public databases were performed at https://www.aclbi.com/.

## 3 RESULTS

### 3.1 Lipocalin‐2 deficiency in mice inhibits the development of hypoxia‐induced PH

**FIGURE 1:**
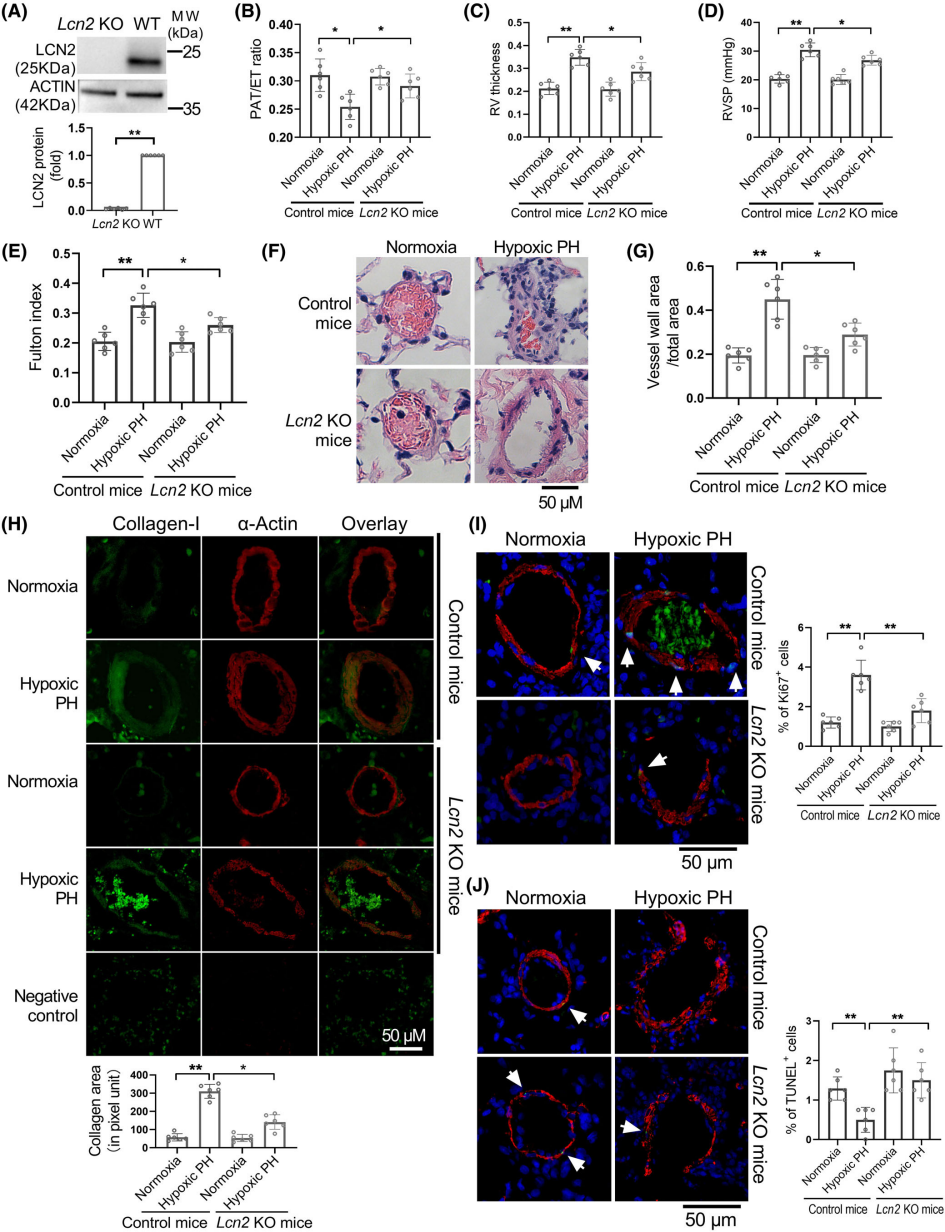
Lipocalin‐2 (LCN2) deficiency in mice attenuates chronic hypoxia induced pulmonary hypertension (PH). (A), Western blot analysis of LCN2 protein levels in lung homogenates of wild type (WT, control) and *Lcn2* knockout (KO) mice. (B and C), WT and *Lcn2* KO mice were exposed to hypoxia (10% O_2_) or room air (normoxia) for 4 weeks. Echocardiography showing the pulmonary artery acceleration time (PAAT) and right ventricular (RV) wall thickness. (D), Changes in RVSP assessed by closed‐chest catheterization. (E), Changes in Fulton index (right ventricle/[left ventricle + septum]). (F), Representative H&E staining of distal pulmonary arteries from control mice and *Lcn2* KO mice exposed to normoxia or hypoxia for 4 weeks. (G), Quantification of distal pulmonary artery thickness as measured by the ratio of vessel wall area to total vessel area. (H), Lung slides were double stained for collagen‐I (green) and α‐Actin (red). Representative image and quantification of collagen‐I level in distal pulmonary artery were shown. (I), Lung slides were double stained for Ki67 (green) and α‐Actin (red). Representative image and quantification of Ki67^+^ cells in distal pulmonary artery were shown. (J), Lung slides were double stained for TUNEL (green) and α‐Actin (red). Representative image and quantification of TUNEL^+^ cells in distal pulmonary artery were shown. Results are expressed as mean ± SE; *n* = 6 mice per group. Statistical significance was determined by Mann–Whitney *U* test (A) or Kruskal–Wallis test (B‐J). * *p* < 0.05 and ** *p* < 0.01.

The *Lcn2* KO (*Lcn2* ^−/−^) mice were used to explore the role of LCN2 in PH progression. The lungs from *Lcn2* KO mice exhibit no LCN2 expression (Figure 1A), confirming successful knockout of the LCN2 gene. *Lcn2* KO mice and control mice were exposed to hypoxia (10% O_2_) for 4 weeks. Haemodynamic analysis showed that hypoxic PH mice have shorter PAAT and higher RVSP, RV wall thickness and Fulton index than normoxic mice. LCN2 deficiency mitigated the reduction in PAAT and the elevation in RVSP, RV wall thickness and Fulton index caused by hypoxia (Figure 1B–E and Figure S1A,B). No significant difference was seen in CO between the mice under hypoxia and normoxia (Figure S1C). Moreover, the hypoxia‐induced enhancements in the thickness of distal pulmonary artery walls, production of collagen‐I protein, SMC proliferation, as well as reductions in SMC apoptosis in pulmonary arterioles, were all alleviated in *Lcn2* KO mice (Figure 1F–J). Together, these results indicate that the deletion of LCN2 mitigates hypoxia‐induced PH and vascular remodelling.

### 3.2 LCN2 deficiency in mouse inhibits the glycolysis of hypoxia‐induced PH

**FIGURE 2:**
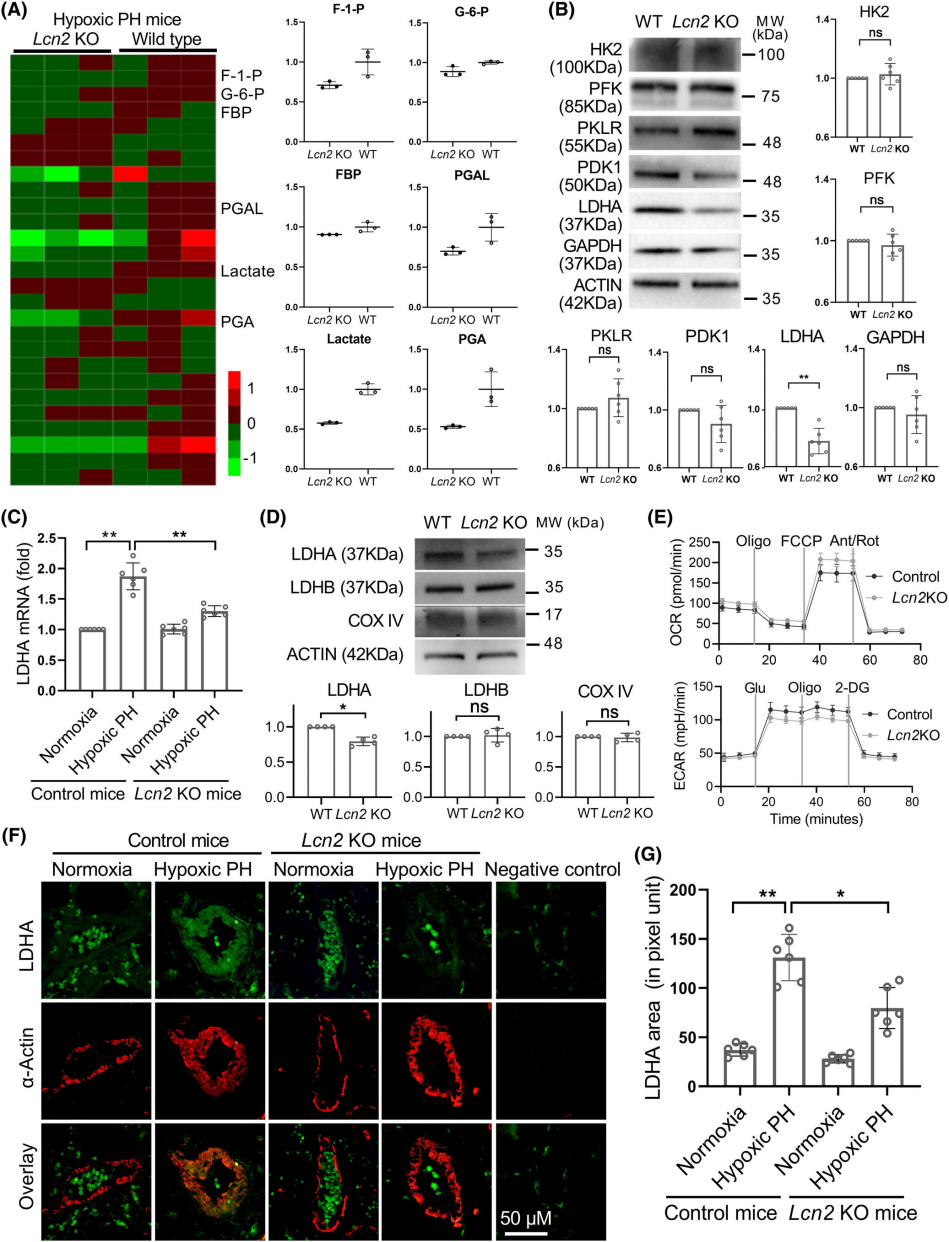
LCN2 deficiency in mice inhibits the glycolysis of hypoxia‐induced PH. A, WT and *Lcn2* KO mice were exposed to hypoxia or normoxia for 4 weeks. Heatmap of metabolome profiles from mouse lung tissue samples. Heatmap colours represent relative metabolite levels as indicated in the colour key. The significantly changed metabolites were shown as histograms. F‐1‐P, fructose‐1‐phosphate; G‐6‐P, glucose‐6‐phosphate; FBP, fructose‐1,6‐phosphate; PGAL, glyceraldehydes‐3‐phosphate; PGA, 3‐phosphoglyceric acid. (B), Western blot and quantification analysis of the levels of glycolytic enzymes in lung homogenates of WT and *Lcn2* KO mice. (C), Real‐time PCR analysis of LDHA mRNA levels in lung homogenates of WT and *Lcn2* KO mice exposed to hypoxia or normoxia. (D), The PASMCs were isolated from *Lcn2* KO and control mice with hypoxic PH. Western blot and quantification analysis of the levels of glycolytic enzymes in isolated PASMCs. (E), Extracellular acidification rate (ECAR) and oxygen consumption rate (OCR) were measured to evaluate the glycolysis and mitochondrial respiration of isolated PASMCs. (F and G), Lung slides were double stained for LDHA (green) and α‐Actin (red). Representative images and quantification of LDHA staining in distal pulmonary artery of WT and *Lcn2* KO mice exposed to hypoxia or normoxia. Results are expressed as mean ± SE. *n* = 6 mice per group (B, C and G), *n* = 3 data point (each data point represents the result of a mixed blood sample from two mice in the same group) (A and E), or *n* = 4, independent experiments (D). Statistical significance was determined by Mann–Whitney *U* test (A, B, D and E) or Kruskal–Wallis test (C and G). * *p* < 0.05 and ** *p* < 0.01, ns *p* > 0.05.

To investigate the role of glucose metabolism in the development of PH promoted by LCN2 in mice, we conducted a study where 27 glucose‐related metabolites were extracted using targeted capillary electrophoresis‐mass spectrometry. We found a strong decline in the levels of glycolytic metabolites in *Lcn2* KO PH mice compared to wild‐type PH controls (Figure 2A). We then examined the expression levels of key glycolytic enzymes, including HK2, PFKM, PKM2, LDHA and PDK1, to explore the underlying reason behind this switch. We observed a notable reduction only in the expression of LDHA in *Lcn2* KO mice compared to control mice in the hypoxia induced PH model (Figure 2B). However, the LDHA expression level was significantly elevated in PH mice compared to controls, regardless of *Lcn2* KO status (Figure 2B,C). In order to elucidate the metabolic changes occurring in the thickened pulmonary arteries, disregarding potential changes in other cell types within lung tissue, the PASMCs were isolated from *Lcn2* KO and control mice with hypoxic PH. As shown in Figure 2D, knockout of *Lcn2* significantly decreased the expression level of LDHA in isolated PASMCs. Additionally, the protein level of LDHB, another crucial subunit of LDH in muscle tissue, showed no significant change. Similarly, the protein level of mitochondrial complex IV, a rate‐limiting enzyme in the mitochondrial oxidative phosphorylation process, did not show significant changes between PASMCs from *Lcn2* KO and control mice. Furthermore, we utilized a Seahorse Extracellular Flux analyser to measure the ECAR and OCR of isolated PASMCs. PASMCs from *Lcn2* KO mice exhibit a significant decrease in ECAR and a significant increase in OCR compared to those from control mice (Figure 2E and Figure S2A,B). In omics analysis based on human patients, previous reports indicated an up‐regulation of LDHA in PAH patients.^29^ Subsequently, an online analysis using publicly available omics data (https://www.ncbi.nlm.nih.gov/geo; GSE113439) revealed that LDHA is significantly up‐regulated and positively correlated with the expression level of LCN2 in PAH patients (Figure S2C,D). Additionally, the expression of LDHA and lactate levels were significantly elevated and correlated with LCN2 expression level in lung homogenates of PH rats (Figure S2E–I). Moreover, the hypoxia‐induced increase in LDHA expression in the distal pulmonary artery wall was significantly reduced in *Lcn2* KO mice (Figure 2F,G). Similar results were observed in rat PH models induced by MCT or sugen/hypoxia (Figure S2J,K). These results suggest that LDHA may play a role in mediating the inhibition of hypoxia‐induced PH in *Lcn2* KO mice.

### 3.3 Heterozygous deficiency of LDHA in mice suppresses the development of hypoxia‐induced PH

**FIGURE 3:**
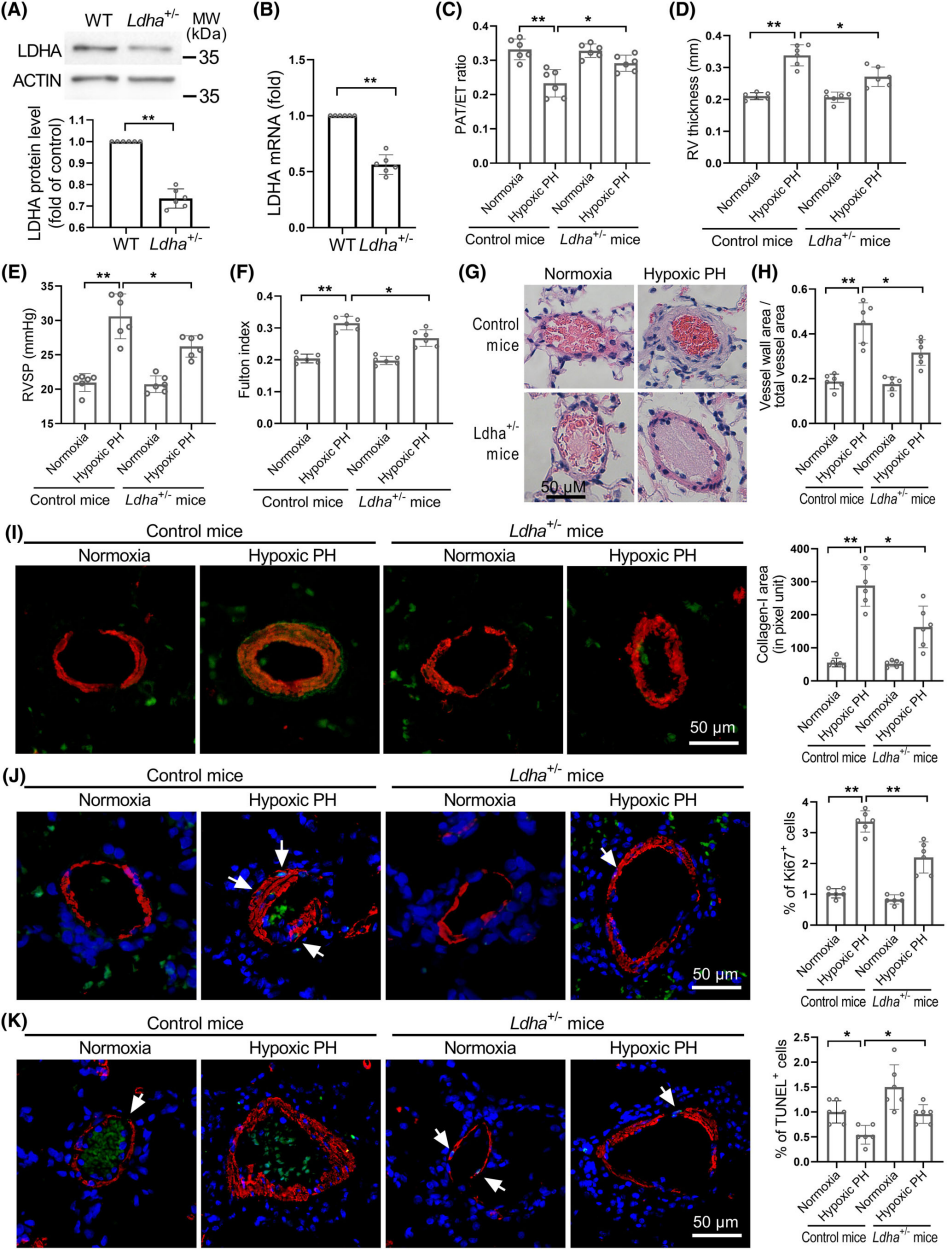
Heterozygous LDHA deficiency in mice inhibits the development of hypoxia‐induced PH. (A and B), Western blot and Real‐time PCR analysis of LDHA expression levels in lung homogenates of wild type (WT, control) and heterozygous *Ldha* knockout (*Ldha* ^+/−^) mice. (C and D), *Ldha* ^*+/−*^ and control mice were exposed to hypoxia or normoxia for 4 weeks. Echocardiography showing the changes in PAAT and RV thickness. (E), Changes in RVSP assessed by closed‐chest catheterization. (F), Changes in Fulton index. (G and H), Representative H&E staining and quantification of distal pulmonary arteries thickness from control mice and *Ldha* ^*+/−*^ mice exposed to normoxia or hypoxia. (I–K), Representative image and quantification of collagen‐I level, Ki67^+^ cells, and TUNEL^+^ cells in distal pulmonary artery were shown. Results are expressed as mean ± SE; *n* = 6 mice per group. Statistical significance was determined by Mann–Whitney *U* test (A and B) or Kruskal–Wallis test (C‐K). * *p* < 0.05 and ** *p* < 0.01.

Due to the embryonic lethality of homozygous *Ldha* knockout mice,^30^ *Ldha* ^+/−^ mice were used to investigate the impact of genetic *Ldha* deficiency on the development of PH. Compared to *Ldha* ^+/+^ (control) mice, the lungs of *Ldha* ^+/−^ mice showed lower levels of LDHA mRNA and protein (Figure 3A,B), indicating successful knockout of LDHA. *Ldha* ^+/−^ and *Ldha* ^+/+^ mice were subjected to hypoxia treatment (10% O_2_) for 4 weeks. As shown in Figure 3C–F and Figure S3A,B, *Ldha* ^+/−^ attenuated hypoxia‐induced decrease in PAAT and increase in RVSP, RV wall thickness and Fulton index. The CO of hypoxic mice was similar to that of control mice (Figure S3C). Additionally, histological examination of mouse lung tissues using H&E staining and immunostainings revealed decreased thickening of pulmonary vascular wall, collagen‐I protein production, proliferation of SMCs and increased apoptosis of SMCs in pulmonary arterioles of *Ldha* ^+/−^ mice (Figure 3G–K). Together, these results suggest that LDHA knockout alleviates hypoxia‐induced PH and vascular remodelling.

### 3.4 LCN2 induced LDHA expression and aerobic glycolysis promotes proliferation and decreases apoptosis in PASMCs

**FIGURE 4:**
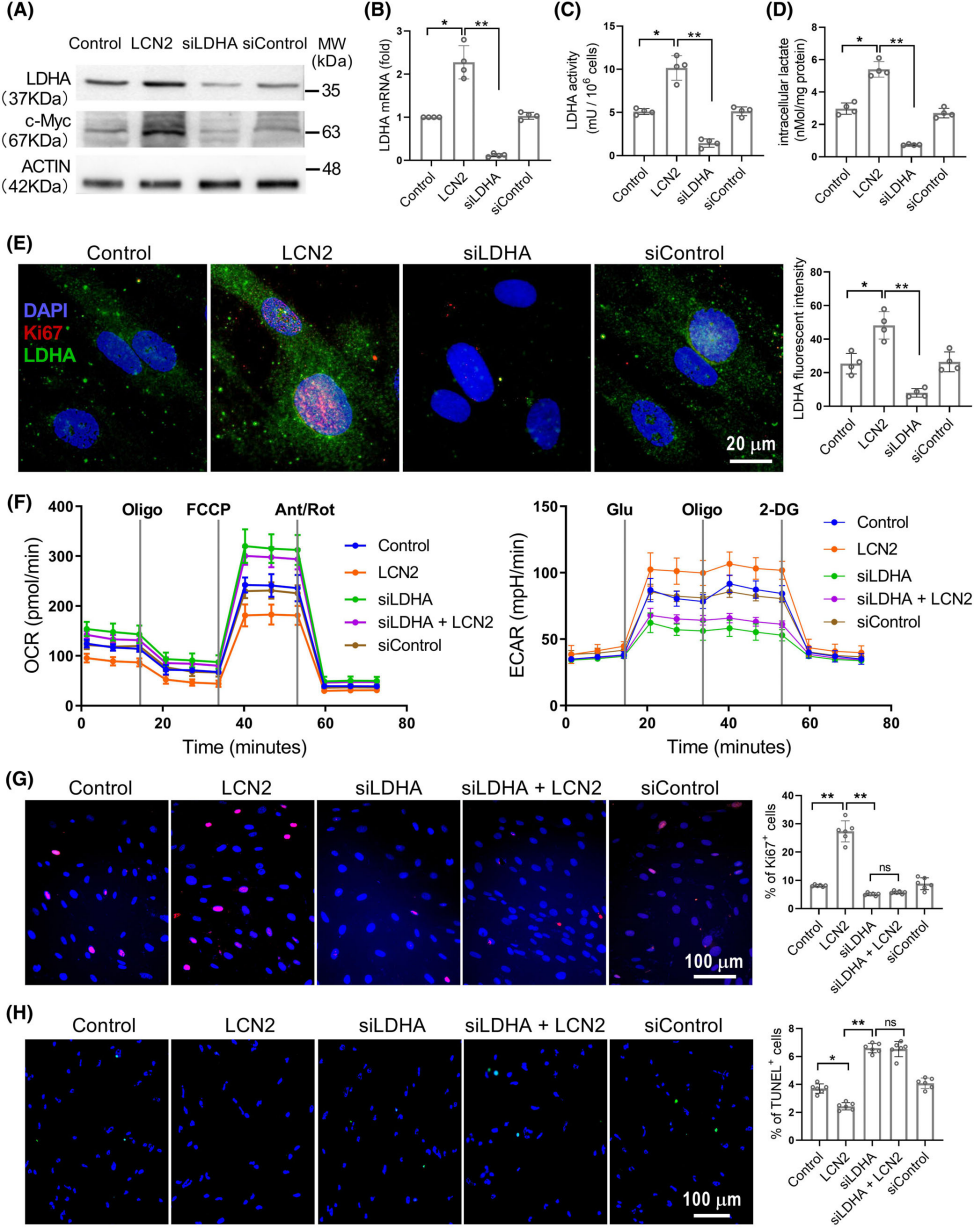
LCN2 induced LDHA expression promotes proliferation and decreases apoptosis in PASMCs. (A–D), In vitro cultured human PASMCs were treated separately with LCN2 (2 nM), LDHA siRNA (siLDHA, 0.1 μM), control siRNA (siControl, 0.1 μM) or saline for 24 h. The protein levels of LDHA and c‐Myc, LDHA mRNA levels, LDHA activity and intracellular lactate levels were measured. (E), Representative images of immunofluorescent staining for LDHA (green), Ki67 (red) and DAPI (blue) to show intracellular LDHA expression. (F), Extracellular acidification rate (ECAR) and oxygen consumption rate (OCR) were measured to evaluate the glycolysis and mitochondrial respiration. (G and H), Representative images and quantification analyses of cell proliferation using Ki67 staining and cell apoptosis using TUNEL assay were shown. Results are expressed as mean ± SE; *n* = 4, independent experiments. Statistical significance was determined by Kruskal–Wallis test. * *p* < 0.05 and ** *p* < 0.01; ns *p* > 0.05.

As shown in Figure 4A–E and Figure S4A,B, incubation of human PASMCs with LCN2 significantly increased LDHA expression and activity. The upstream transcription factor of LDHA, c‐Myc, was also up‐regulated by LCN2 treatment. The effect of LCN2 on LDHA expression was further examined in PASMCs under hypoxic conditions. Results showed that co‐culturing with LCN2 led to increased levels of LDHA expression in PASMCs under both hypoxic and normoxic conditions (Figure S4C). Interestingly, incubation of PASMCs with LCN2 significantly increased the ECAR and decreased the OCR in the Seahorse Extracellular Flux analyzes. LDHA siRNA (Figure 4A–E and Figure S4A,B) eliminated the LCN2‐induced increase in ECAR and decrease in OCR (Figure 4F and Figure S4D,E). These results suggest that LCN2‐induced LDHA expression promotes aerobic glycolysis in cultured human PASMCs. We have previously reported that LCN2 enhances proliferation and reduces apoptosis in cultured PASMCs.^24^, ^25^, ^26^ In this study, the LCN2‐induced proliferation and reduced apoptosis were also abolished by LDHA knockdown (Figure 4G,H). These results indicate that LDHA is a critical downstream mediator of LCN2 signalling in PASMCs.

### 3.5 LCN2 enhances LDHA expression through activation of the PI3K/Akt pathway in PASMCs

**FIGURE 5:**
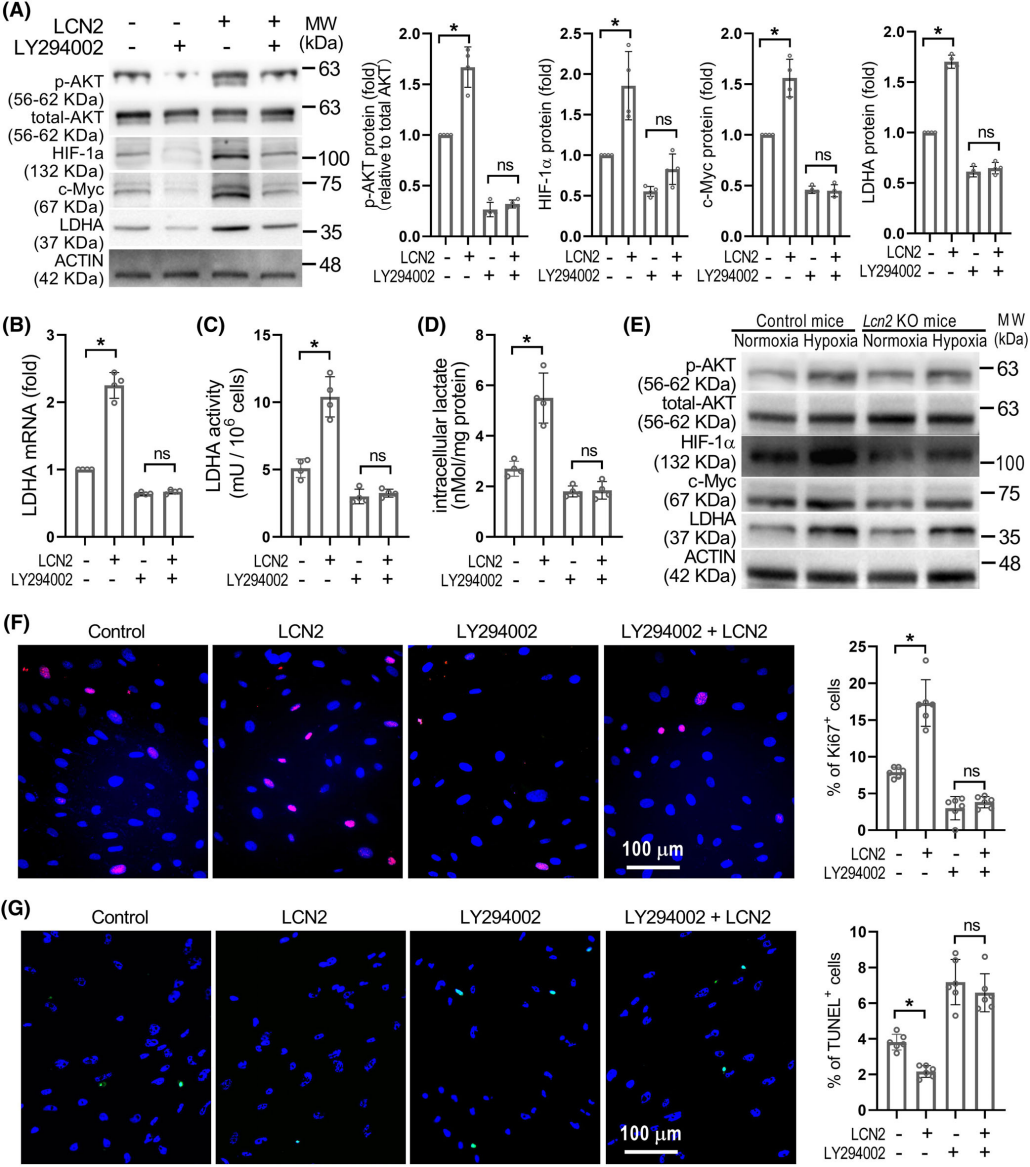
LCN2 promotes LDHA expression via activation of PI3K/Akt pathway. (A–D), in vitro cultured human PASMCs were treated with LCN2 (2 nM), Ly294002 (20 μM) or saline for 24 h. The Representative Western blot images of LDHA, c‐Myc, total Akt and phosphorylated Akt, LDHA mRNA levels, LDHA activity and intracellular lactate levels were shown. *n* = 4, independent experiments. (E), Representative Western blot images of LDHA, c‐Myc, HIF‐1α and Akt in lung homogenates of *Lcn2* KO and control mice exposed to hypoxia or normoxia were shown; *n* = 6 mice per group. (F and G), Representative images and quantification of cell proliferation using Ki67 staining and cell apoptosis using TUNEL assay were shown; *n* = 4, independent experiments. Results are expressed as mean ± SE. Statistical significance was determined by Kruskal–Wallis test. * *p* < 0.05 and ns *p* > 0.05.

We have previously reported that LCN2 is capable of activating the PI3K/Akt signalling pathway in PASMCs,^25^ and this pathway has been implicated in the progression of PH.^31^ To explore whether LCN2 up‐regulated LDHA expression is mediated through PI3K/Akt signalling, we investigated the effects of the PI3K/Akt inhibitor (LY294002). As shown in Figure 5A–D, inhibition of PI3K/Akt signalling with LY294002 in cultured PASMCs effectively counteracted the LCN2‐induced elevation in LDHA expression and activity. Furthermore, the enhanced proliferation and reduced apoptosis induced by LCN2 were nullified by LY294002 treatment in PASMCs (Figure 5F,G). In lung tissue from MCT rats and sugen/hypoxia rats, there was a notable increase in the expression levels of phosphorylated Akt (p‐AKT), mirroring the changes observed in LCN2 and LDHA expression (Figure S5A–H). Interestingly, the hypoxia‐induced phosphorylation of Akt, as well as the expression of LDHA and cMyc in the distal pulmonary artery wall, was significantly diminished in *Lcn2* KO mice (Figure 5E and Figure S6A–F). These results indicate that LCN2 boosts LDHA expression through the PI3K/Akt pathway in PASMCs.

### 3.6 LCN2 promotes LDHA expression through the PI3K/Akt‐HIF‐1α pathway in PASMCs

**FIGURE 6:**
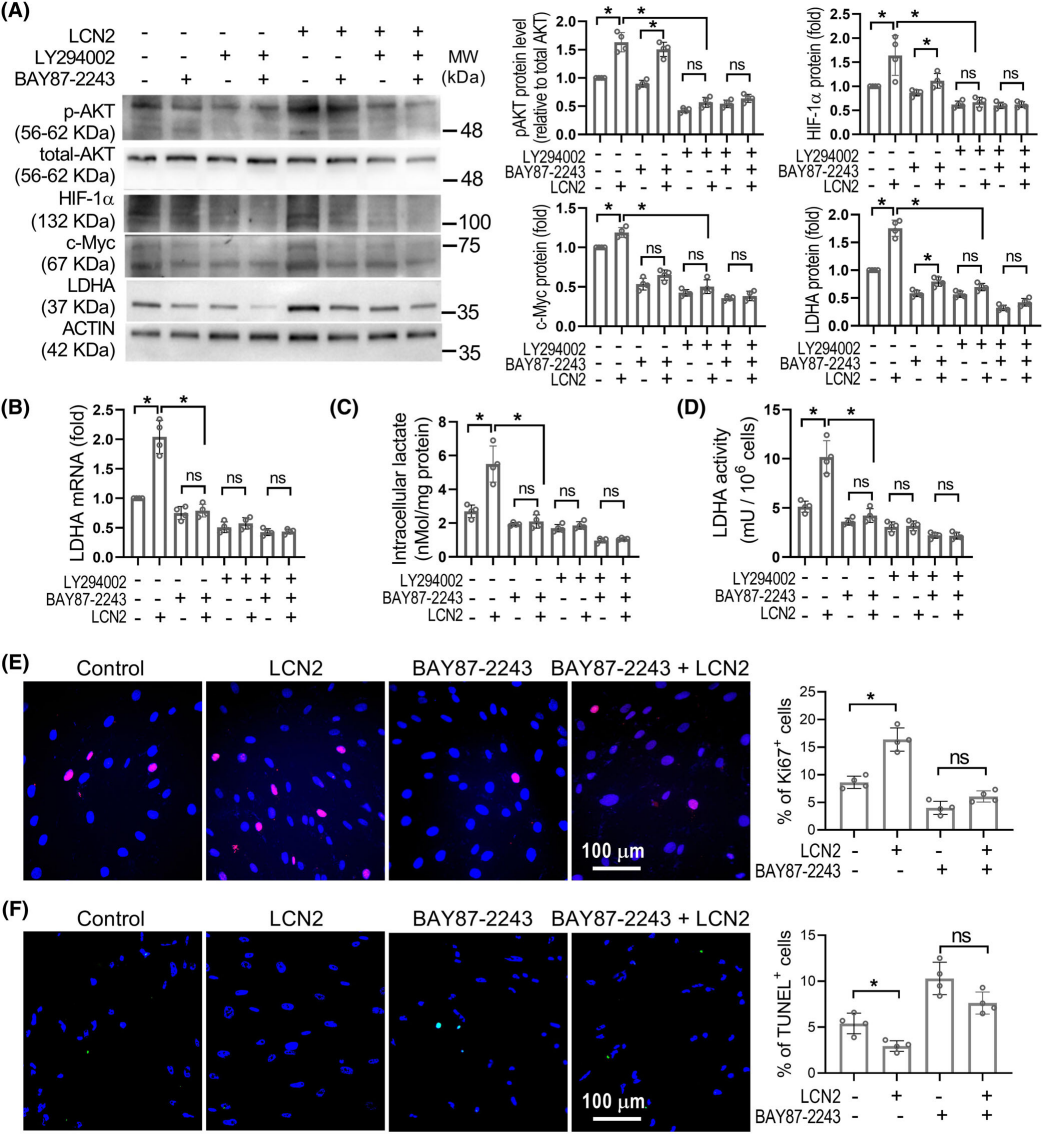
LCN2 promotes LDHA expression via activation of HIF‐1α. (A), in vitro cultured human PASMCs were treated with LCN2 (2 nM), Ly294002 (20 μM), BAY87‐2243 (20 nM) or saline for 24 h. The representative Western blot images and quantification of LDHA, c‐Myc, HIF‐1α, total Akt and phosphorylated Akt were shown. The lanes represented came from the same blot. (B), Quantification analyss of the LDHA mRNA levels were shown. (C and D), Quantification analyses of the intracellular lactate levels and LDHA activity were shown as histograms. (E and F), Representative images and quantification of cell proliferation using Ki67 staining and cell apoptosis using TUNEL assay were shown. Results are expressed as mean ± SE. *n* = 4, independent experiments. Statistical significance was determined by Kruskal–Wallis test. * *p* < 0.05 and ns *p* > 0.05.

It was reported that the transcription factor HIF‐1α mediates hypoxia‐induced LDHA expression in glioblastoma cells^32^ and is an important pathological factor in PH, particularly in hypoxia‐induced PH.^33^ We then investigated whether HIF‐1α mediates the LCN2 up‐regulated LDHA expression in cultured human PASMCs. As shown in Figure 6A, similar to the changes observed in p‐AKT and LDHA levels, LCN2 treatment resulted in an elevation of HIF‐1α expression in PASMCs. The increase in HIF‐1α expression induced by LCN2 could be reversed by LY294002. When HIF‐1α was inhibited by BAY87‐2243 in LCN2‐stimulated PASMCs, the protein levels of p‐AKT and HIF‐1α remained elevated, while the increase in LDHA protein level and activity was abolished (Figure 6A–D). Likewise, suppression of HIF‐1α nullified the effects of LCN2 on PASMC proliferation and apoptosis (Figure 6E,F). Under normoxia conditions, HIF‐1α is maintained at a low level due to the hydroxylation of its proline residues by HIF‐1α‐specific prolyl hydroxylases, leading to its degradation via proteasomes. We also assessed the hydroxylation level of HIF‐1α to confirm the regulatory influence of LCN2 on HIF‐1α. The results showed that following LCN2 treatment, the proline hydroxylation level of HIF‐1α decreased significantly, correlating with the elevation in its protein level (Figure S7). Notably, HIF‐2α did not exhibit significant changes in protein levels after LCN2 treatment.

**FIGURE 7:**
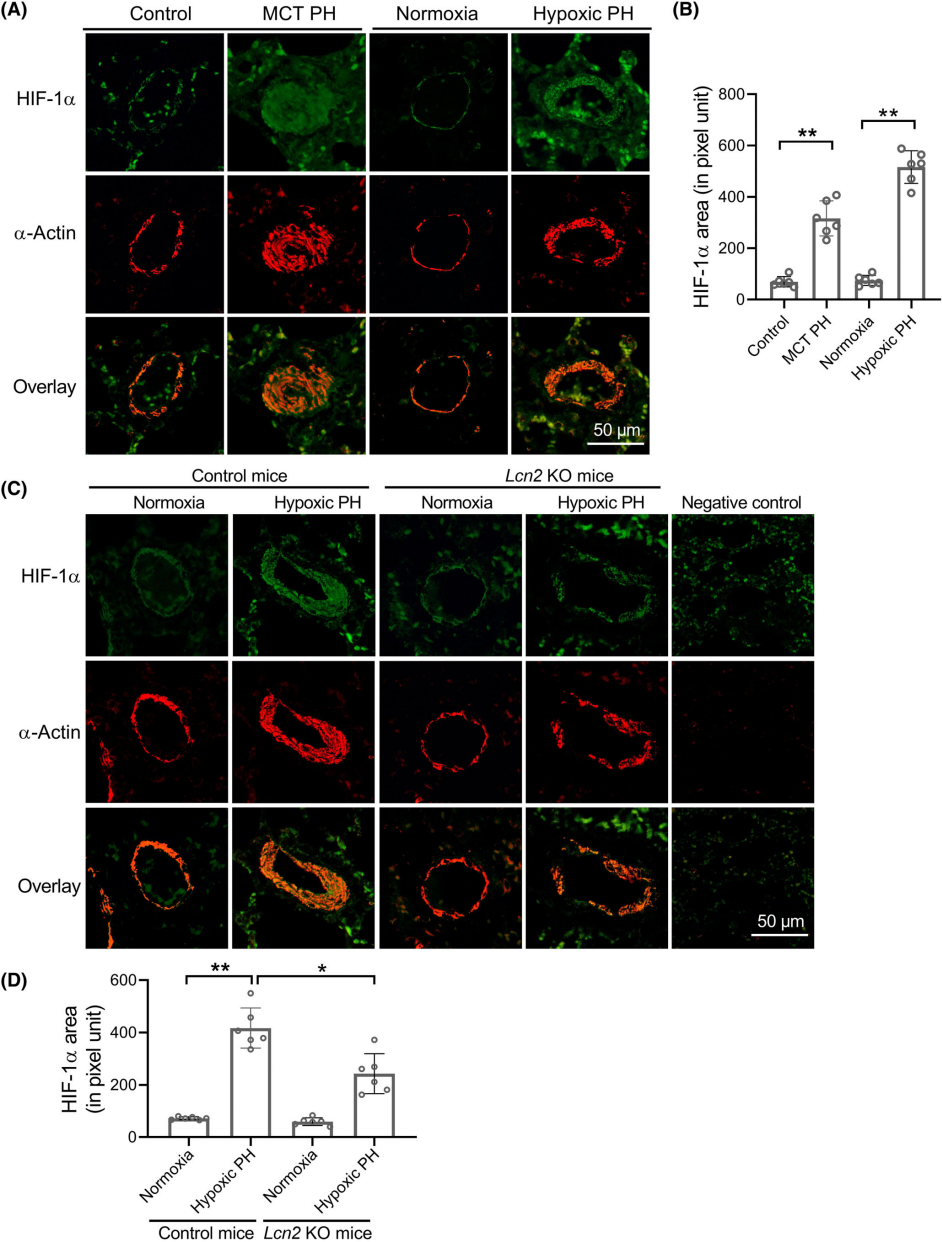
LCN2 deficiency in mice attenuates chronic hypoxia induced HIF‐1α expression in lung tissue. (A and B), Representative images and quantification analysis of HIF‐1α staining in distal pulmonary artery of MCT PH, hypoxic PH and control rats. (C and D), Wild type (control) and *Lcn2* KO mice exposed to hypoxia (10% O_2_) or room air (normoxia) for 4 weeks. Representative images and quantification analysis of HIF‐1α staining in distal pulmonary artery of control or *Lcn2* KO mice were shown. Results are expressed as mean ± SE; *n* = 6 mice per group. Statistical significance was determined by Mann–Whitney *U* test (B) or Kruskal–Wallis test (D). * *p* < 0.05 and ** *p* < 0.01.

In lung tissue from MCT rats and sugen/hypoxia rats, the expression levels of HIF‐1α were up‐regulated and correlated with LCN2, p‐AKT and LDHA levels (Figure 7A,B and Figure S5). In *Lcn2* KO mice, the hypoxia‐induced up‐regulation of HIF‐1α in the distal pulmonary artery wall was significantly reduced compared to control mice (Figure 5E, Figure 7C,D and Figure S6B). These results indicate a signalling axis involving LCN2‐pAKT‐HIF1α‐LDHA in PASMCs of PH.

## 4 DISCUSSION

Our previous studies have reported that LCN2 was up‐regulated in PH and could promote the proliferation of human PASMC.^24^, ^25^, ^26^ Nevertheless, the intricate role of LCN2 in the pathogenesis and progress of PH remained unclear. In the present study, we have elucidated a pivotal function of LCN2 in glycolytic regulation, which forms the fundamental basis of the metabolic theory in the progression of PH. Our data show that LCN2 significantly contributes to glycolysis‐related collagen synthesis and PASMC proliferation through pAkt‐HIF‐1α‐stimulated LDHA expression. Our data underscore the transformative potential of LCN2 and its signalling pathway in the treatment of PH.

A large number of studies suggest that LCN2 is a carcinogenic protein implicated in tumour initiation, advancement and metastasis.^18^, ^19^, ^20^, ^21^, ^22^ Recent reports have highlighted the involvement of LCN2 in metabolic regulatory.^27^, ^28^ In the present study, using *Lcn2* KO mice, we have established that LCN2 deficiency markedly inhibits hypoxia‐induced haemodynamic alterations and pulmonary artery remodelling, underscoring its critical role in the progression of PH. Our results urge further exploration into the potential role of LCN2 in metabolic abnormalities associated with pulmonary hypertension. Metabolomic analysis revealed a significant decline in glycolytic metabolites in *Lcn2* KO PH mice compared to wild‐type PH controls. Additionally, assessment of the protein levels of key glycolytic enzymes demonstrated that LCN2 deficiency notably reduced the expression level of LDHA in hypoxia induced PH mice. Notably, LDHA and its metabolic by‐product lactic acid are substantially up‐regulated in various rodent PH models, with reports indicating that LDHA up‐regulation can stimulate tumour progression.^13^, ^14^, ^15^, ^16^ Our results showcased that partial LDHA deficiency effectively inhibited the progression of hypoxic‐induced PH in mice. Moreover, analysis of publicly available omics data revealed a significant up‐regulation of LDHA in human PAH patients, consistent with prior research.^29^ Therefore, we propose that the down‐regulation of LDHA facilitates the inhibitory effect of LCN2 deficiency on PH progression. In our present study, incubation of PASMCs with LCN2 resulted in a marked elevation in LDHA expression and activity, including augmented aerobic glycolysis and proliferation, along with reduced apoptosis. Given the substantial up‐regulation of LCN2 expression in both PH animal models and human patients,^23^, ^24^ we posit that LCN2‐mediated LDHA expression plays a pivotal role in the advancement of PH.

LDHA catalysed reactions primarily lead to the production of lactate, serving as the ultimate driver of the Warburg effect in cancer cells.^7^ In the metabolic framework of PH, it is postulated that escalated glycolysis and heightened lactate levels instigate the remodelling of pulmonary vascular.^1^ A recent study reports that lactate could induce collagen synthesis and cellular proliferation of cultured PASMCs.^34^ However, the precise mechanisms through which lactate induces the remodelling of pulmonary arteries deserve further study. In cancer cells, deregulated LDHA facilitates the regeneration of NADH into NAD^+^ and boosts metabolic flux through the pentose phosphate pathway, and hence fuelling cancer cell growth by providing essential metabolic substrates.^7^ Likewise, in the context of PH, an observation indicates that elevated NADH levels may promote the proliferative of adventitial fibroblasts.^4^ The accumulation of lactate caused by LDHA up‐regulation might also exert its influence through protein post‐translational modifications. Notably, emerging research by Chen and colleagues reported that lactate accumulation can induce histone lactylation, with LDH inhibitor mitigating histone lactylation, thereby ameliorating PASMC proliferation and vascular remodelling in hypoxic PH rats.^5^

To date, the regulation of LDHA expression in PH remains unexplored. Dysregulated LDHA expression in various diseases is attributed to a myriad of mechanisms. Activation of LDHA expression can be facilitated by the transcription factors c‐Myc^14^, ^35^, ^36^, ^37^ or HIF‐1^38^, ^39^ in different tumour contexts. Furthermore, c‐Myc and HIF‐1 have been shown to cooperatively enhance LDHA transcription in cancer cells.^40^ Both c‐Myc^41^, ^42^ and HIF‐1^43^, ^44^, ^45^ have been reported to be up‐regulated in PH. In a previous study, we documented that LCN2 facilitates PASMC proliferation via activation of the PI3K/Akt signalling pathway.^25^ In the present study, we unveil that both Akt phosphorylation and HIF‐1α activation are implicated in LCN2‐mediated LDHA transcription regulation. Notably, inhibition of HIF‐1α using a small molecule inhibitor in cultured PASMCs abolished the upregulation of LDHA induced by LCN2, while the protein levels of p‐Akt and HIF‐1α remained elevated. Moreover, chronic hypoxia‐induced up‐regulation of HIF‐1α and p‐Akt in the distal pulmonary artery wall of *Lcn2* KO mice was significantly attenuated compared to control mice. Therefore, our results delineate a signalling cascade involving LCN2‐pAkt‐HIF‐1α‐LDHA in PASMCs, underscoring its critical role in PH. It is important to acknowledge that our study does not exclude the possibility of other unidentified mechanisms mediating the promotive impact of LCN2 on LDHA expression.

As a survival‐promoting protein, LCN2 exerts diverse biological effects, such as promoting inflammation,^46^, ^47^ inhibiting apoptosis^20^, ^21^ and reducing autophagy.^48^, ^49^ In our previous study, we have demonstrated that LCN2 can enhance proliferation and reduce apoptosis of human PASMCs.^24^, ^25^, ^26^ Recent studies report that LCN2 is involved in the regulation of glucose metabolism.^19^, ^28^, ^50^ In the present study, LCN2 facilitates the aerobic glycolysis by activating the pAkt‐HIF‐1α‐LDHA pathway in PASMCs. LCN2‐activated Akt signalling has also been observed in cancer cells.^51^, ^52^ Given the prevalent local hypoxia in most solid tumours, LCN2 may play pivotal roles in hypoxic glycolysis in cancer cells as well. In the hypoxic PH model of this study, the PH related parameters and glycolysis markers were notably suppressed in *Lcn2* KO mice compared to control mice, highlighting LCN2's promotion of glycolysis under both aerobic and hypoxic conditions.

LCN2 exists in two forms, with the iron‐free form exhibiting distinct functions from the iron‐loaded form.^53^ While an increase in intracellular iron can lead to the degradation of HIF‐1α, the stability of HIF‐1α is bolstered under hypoxic conditions.^54^, ^55^ Additionally, LCN2 has been reported to perform intracellular function independent of iron,^56^ raising questions about the potential role of iron in LCN2‐mediated aerobic glycolysis in PASMCs. Further investigations into the impact of both iron‐loaded and iron‐free LCN2 on intracellular energy metabolism are warranted.

Beyond PASMCs, pulmonary arterial endothelial cells and adventitial fibroblasts are also involved in the pathogenesis of PH, each exhibiting metabolic abnormalities.^3^, ^4^ Further studies should explore the role of LCN2 in these different pulmonary vascular cell types and in tissue‐infiltrating inflammatory cells in PH.

In conclusion, we demonstrated that up‐regulated LCN2 promotes cell proliferation by enhancing aerobic glycolysis in pulmonary arterial SMCs, thereby contributing to vascular remodelling in PH. This effect is mediated through the activation of pAkt‐HIF‐1α signalling, leading to augmented LDHA expression.

## AUTHOR CONTRIBUTIONS

Conception, design and experiment: G.W., S.L., X.K., H.J., Z.G., M.Z., X.G. and W.L. Analysis and interpretation: G.W., S.L., F.T., N.R., W.L., L.Q. and Y.W. Drafting the manuscript for important intellectual content: G.W., S.L., X.K., F.T., L.Q. and Y.W. All authors reviewed the manuscript.

## FUNDING INFORMATION

This work was supported by the CAMS Innovation Fund for the Medical Sciences (Grant number 2016‐I2M‐1‐015); by the National Natural Science Foundation of China (Grant number 81470424); and by the Natural Science Cultivation Fund of Capital Medical University, china (Grant number PYZ2017104).

## CONFLICT OF INTEREST STATEMENT

The authors declare that we have no known competing financial interests or personal relationships that could have appeared to influence the work reported in this paper.

## Supporting information


**Data S1.** Supporting information.

## Data Availability

The data that support the findings of this study are available from the corresponding author upon reasonable request.This paper does not include the original code.
