# Supplementary material for: Lipocalin‐2 induced LDHA expression promotes vascular remodelling in pulmonary hypertension
Source: Cell Prolif. 2024 Jul 18;57(12):e13717. doi: 10.1111/cpr.13717 (PMC11628741; doi:10.1111/cpr.13717)
Supplement: Supplementary file 1 — Data S1. Supporting information. [file CPR-57-e13717-s001.docx]

**Supplementary Information**

**Lipocalin-2 induced LDHA expression promotes vascular remodeling in pulmonary hypertension**

Guoliang Wang, Shenghua Liu, Xiaohui Kong, Hong Jiao, Feng Tong, Zhangke Guo, Meng Zhang, Xiaoxing Guan, Na Ren, Wanzhen Li, Lihua Qi, Yingjie Wei

**Correspondence:**

[weiyingjie@fuwaihospital.org](mailto:weiyingjie@fuwaihospital.org) (Y.W.), [qiqi76911@bjmu.edu.cn](mailto:qiqi76911@bjmu.edu.cn) (L.Q.) and [wgl163@126.com](mailto:wgl163@126.com) (G.W.)


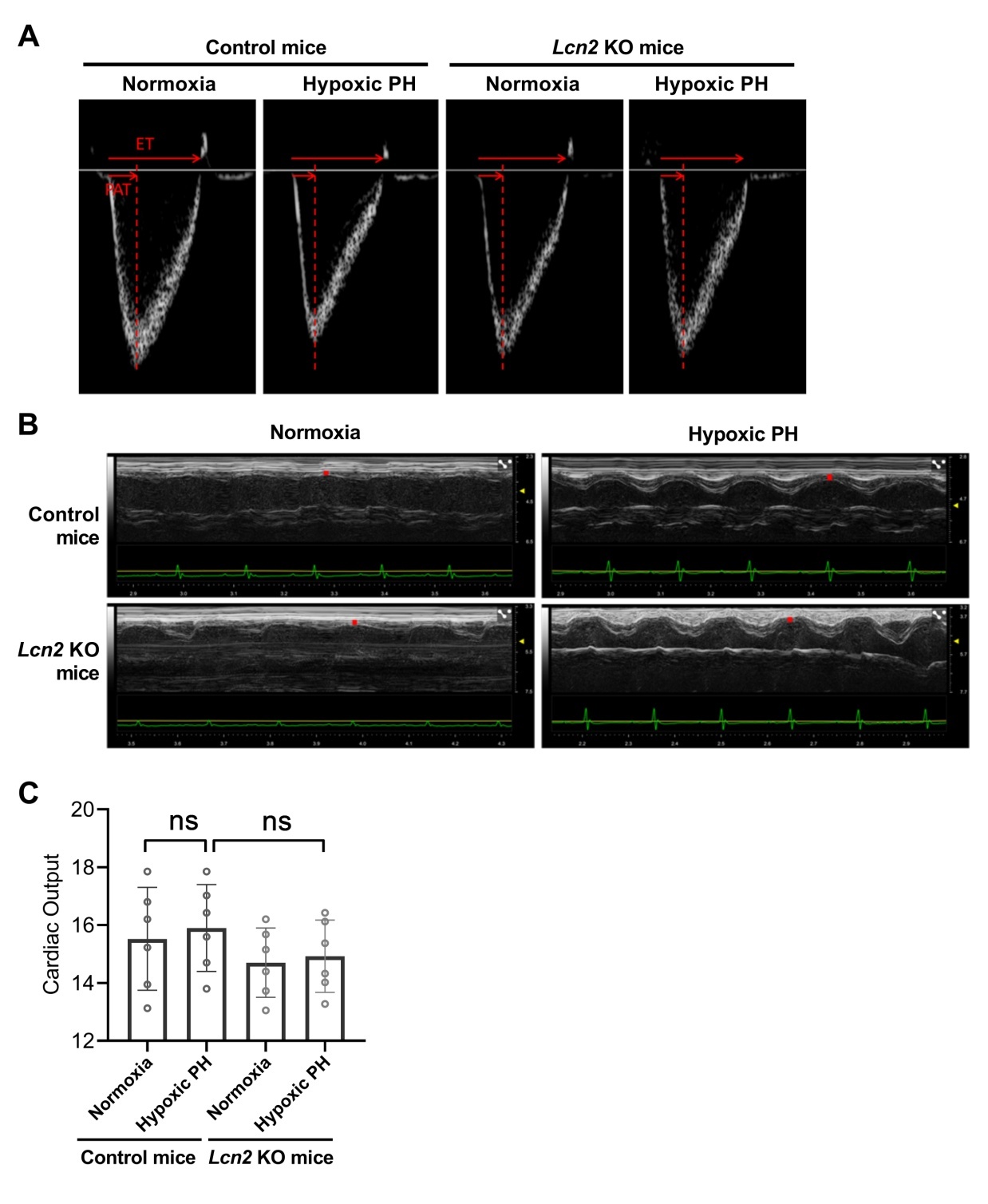


**Figure S1. Lipocalin-2 (LCN2) deficiency in mice attenuates chronic hypoxia induced pulmonary hypertension (PH). (A)** Wild type (control) and *Lcn2* KO mice exposed to hypoxia (10% O_2_) or room air (normoxia) for 4 wk. Representative echocardiography images of the pulmonary artery acceleration time (PAAT). **(B)** Representative echocardiography images of the right ventricular (RV) wall were shown. The short red lines indicate the RV wall thickness during diastole. **(C**) Quantification of cardiac output. Results are expressed as mean ± SE; n=6 mice per group. Statistical significance was determined by Kruskall Wallis test. ns, *P* > 0.05.

**
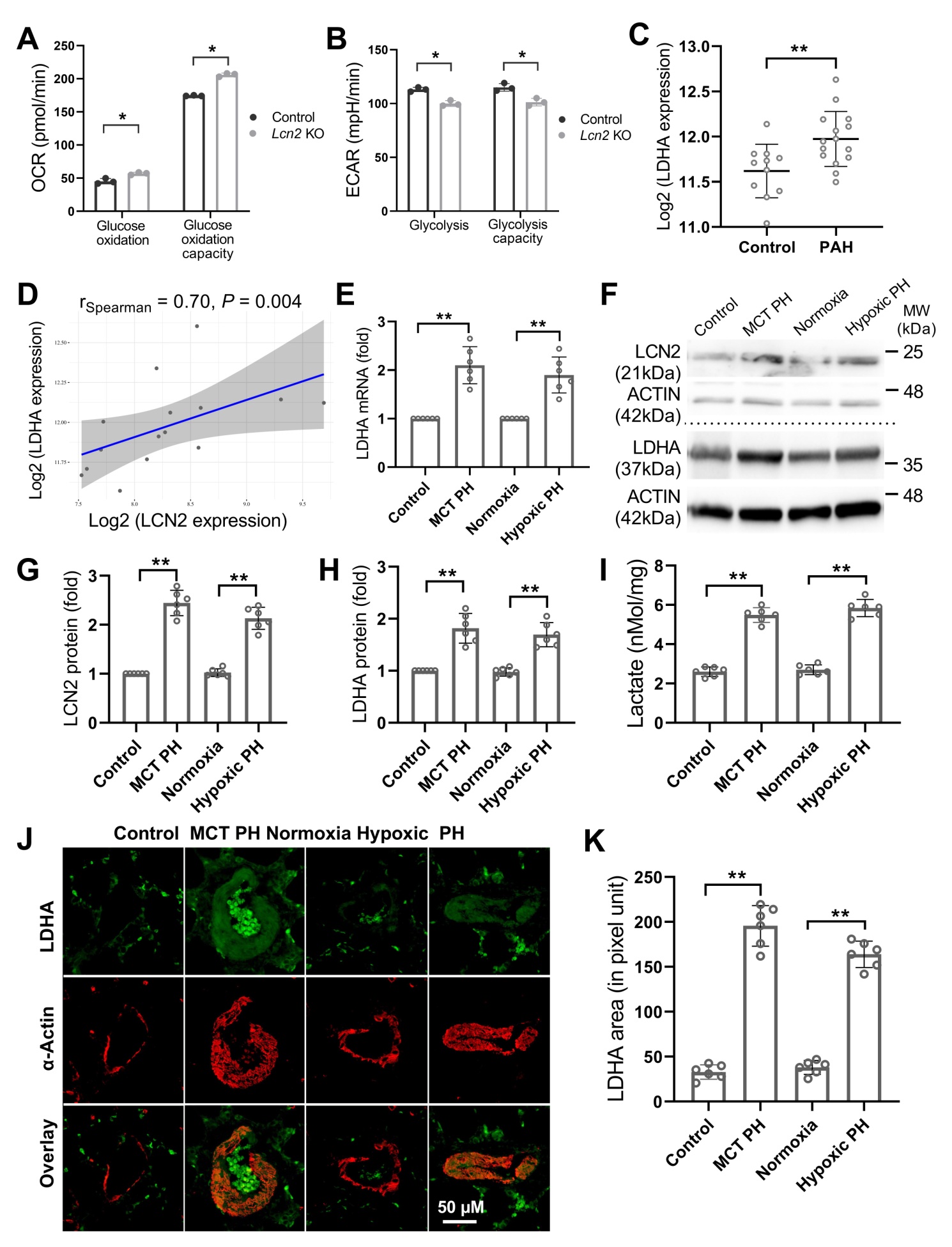
**

**Figure S2. Increased LDHA expression in PH patients and PH rodents. (A** and **B**) The pulmonary artery smooth muscle cells (PASMCs) were isolated from *Lcn2* KO and control mice with hypoxic PH. Extracellular acidification rate (ECAR) and oxygen consumption rate (OCR) were measured to evaluate the glycolysis and mitochondrial respiration of isolated PASMCs (n=3, independent experiments; each point in the scatter plot is the mean of 6 data points from the same set). (**C** and **D**) The expression level of LDHA and its correlation with LCN2 in publicly available omics data (https://www.ncbi.nlm.nih.gov/geo; GSE113439) were analyzed on online platform (https://www.aclbi.com/). (**E**-**H**) Male Sprague-Dawley rats were administered to single subcutaneous injection of monocrotaline (MCT) or saline. Another group of male Sprague-Dawley rats were injected subcutaneously with SU5416 and were exposed to hypoxia for 3 weeks. Real-time PCR and Western blot analysis of LDHA levels in lung homogenates of PH and control rats were shown. (**I)** Quantification of the lactate levels of lung homogenates of PH and control rats. **(J** and **K**) Lung slides were double stained for LDHA (green) and α-actin (red). Representative images and quantification of LDHA staining in distal pulmonary artery of PH and control rats. Results are expressed as mean ± SE; n=6 rats per group (E-K). Statistical significance was determined by Mann Whitney U test. * *P* < 0.05 and ** *P* < 0.01.


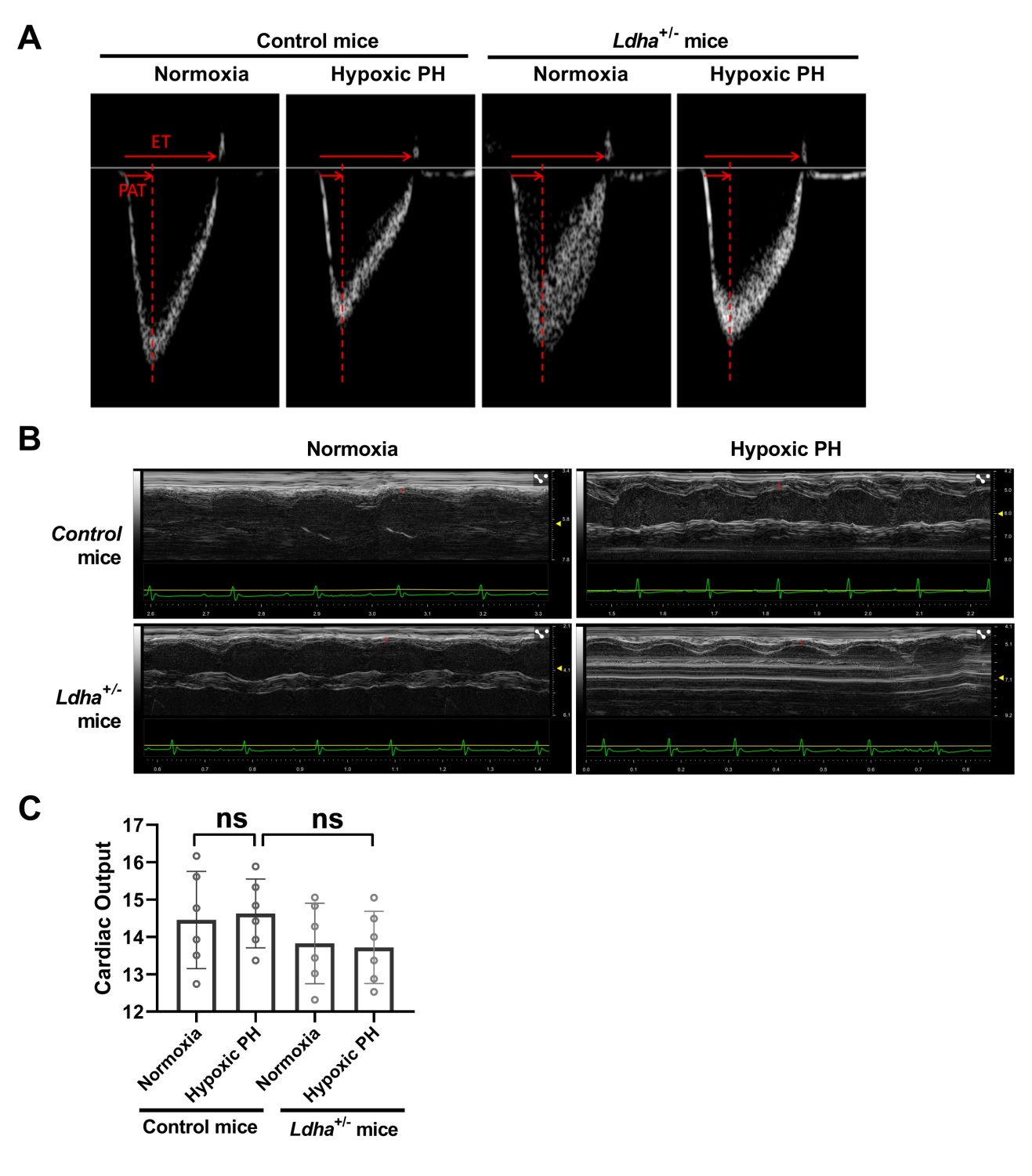


**Figure S3. Heterozygous LDHA deficiency in mice inhibits the development of hypoxia-induced PH.** (A) Wild type (*Ldha*^+/+^) and heterozygous LDHA deficiency (*Ldha*^+/-^) mice exposed to hypoxia (10% O_2_) or room air (normoxia) for 4 wk. Representative echocardiography images of the PAAT. **(B)** Representative echocardiography images of the RV wall were shown. The short red lines indicate the RV wall thickness during diastole. **(C**) Quantification of cardiac output. Results are expressed as mean ± SE; n=6 mice per group. Statistical significance was determined by Kruskall Wallis test. ns, *P* > 0.05.


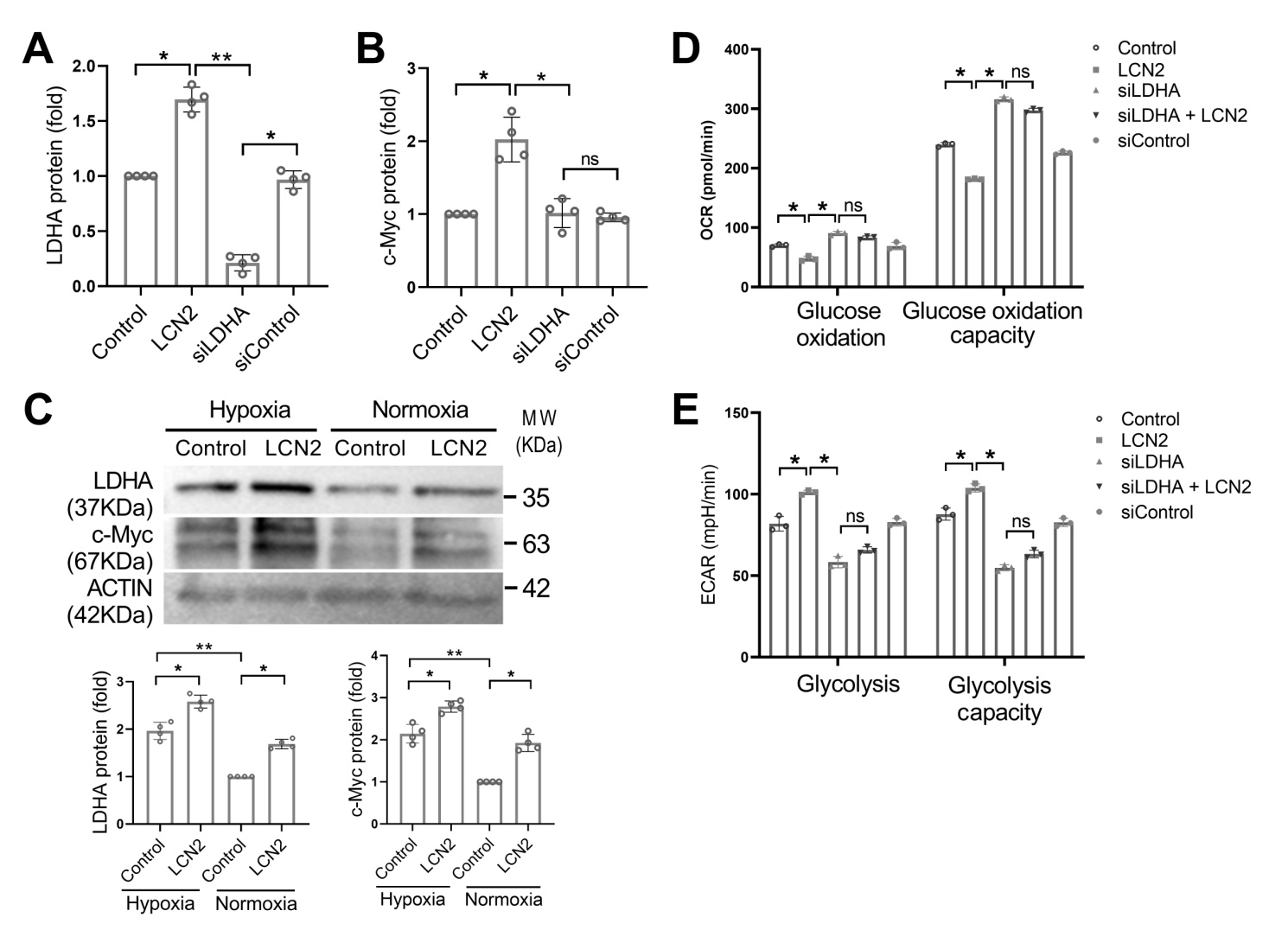


**Figure S4. LCN2 induced LDHA expression and aerobic glycolysis in PASMCs. (A - C**) In vitro cultured human PASMCs were treated with LCN2 (2 nM), LDHA siRNA (0.1 μM), control siRNA (0.1 μM) or saline for 24 hour under normoxia (A and B) or hypoxia (C) conditions. Representative images and quantification analysis of the protein level of LDHA and c-Myc through Western blot was shown (n=4, independent experiments). **(D** and **E**) The oxygen consumption rate (OCR) and extracellular acidification rate (ECAR) were quantified to evaluate the glycolysis and mitochondrial respiration (n=3, independent experiments; each point in the scatter plot is the mean of 5-6 data points from the same set). Results are expressed as mean ± SE. Statistical significance was determined by Kruskall Wallis test. * *P* < 0.05 and ns *P* > 0.05.


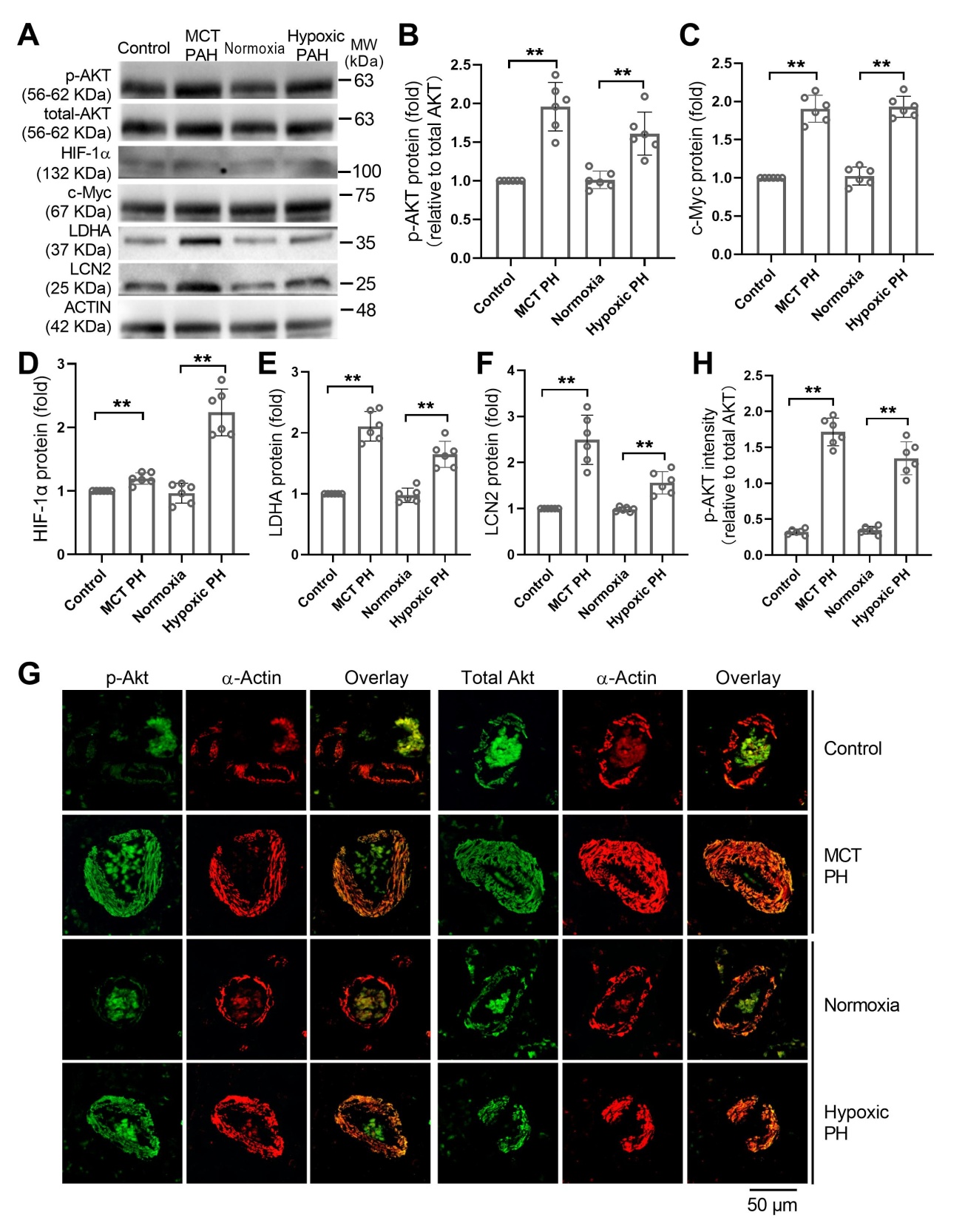


**Figure S5. Increased Akt phosphorylation in distal pulmonary artery of PH rats.** (**A**-**F**) Representative Western blot images and quantification analyses of the protein levels of p-Akt, c-Myc, HIF-1α, LDHA and LCN2 in lung homogenates of PH and control rats were shown. (**G** and **H**) Representative images and quantification analysis of Akt staining in distal pulmonary artery of MCT PH, hypoxic PH and control rats were shown. Results are expressed as mean ± SE; n=6 rats per group. Statistical significance was determined by Mann Whitney U test. ** *P* < 0.01.

**
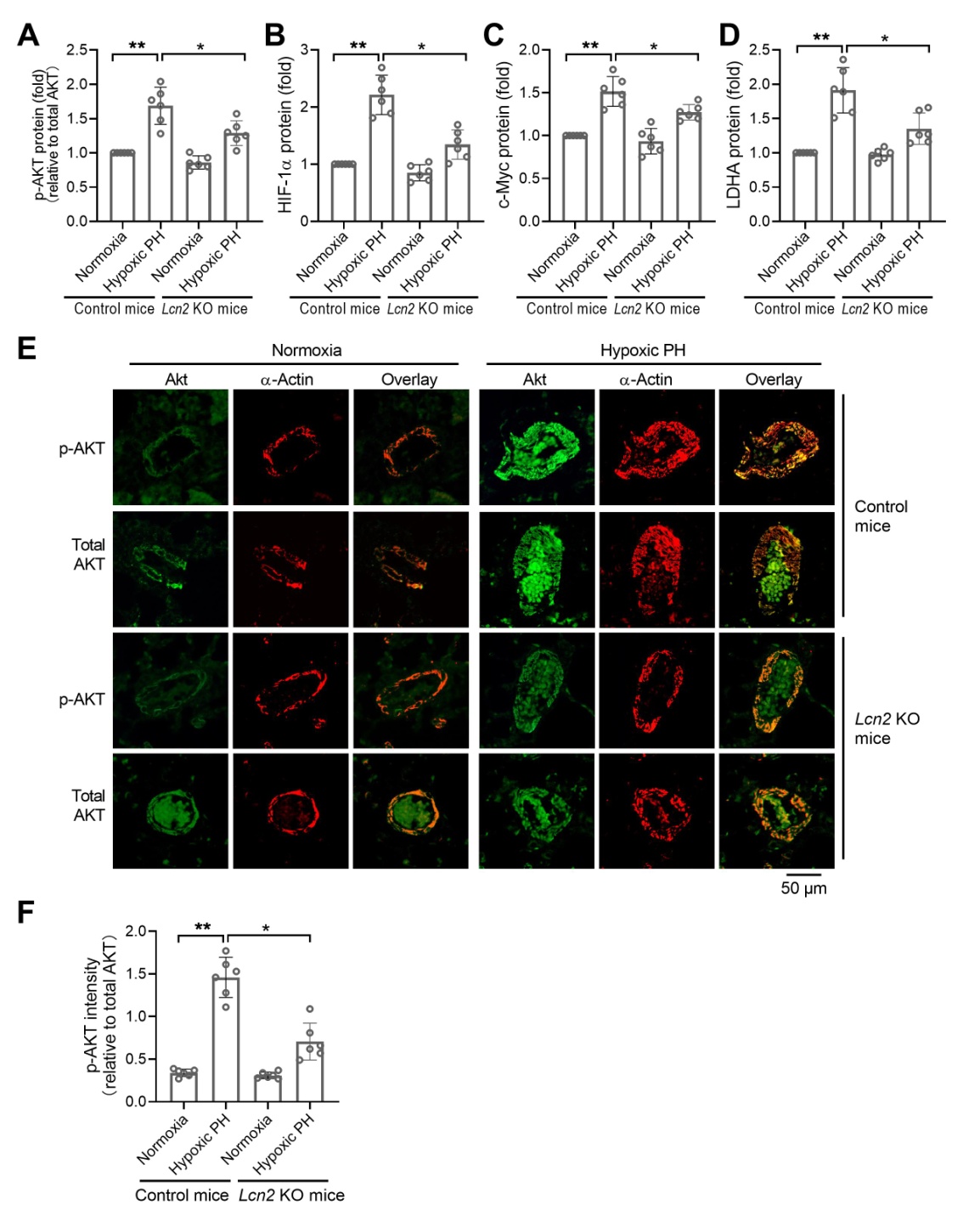
**

**Figure S6. LCN2 deficiency in mice attenuates chronic hypoxia induced Akt phosphorylation and LDHA expression in lung tissue.** (**A**-**D**) Quantification analyses of the protein levels of p-Akt, HIF-1α, c-Myc and LDHA in lung homogenates through Western blot were shown as histograms. (**E** and **F**) Wild type (control) and *Lcn2* KO mice exposed to hypoxia (10% O_2_) or room air (normoxia) for 4 wk. Representative images and quantification analysis of Akt staining in distal pulmonary artery of control or *Lcn2* KO mice were shown. Results are expressed as mean ± SE; n=6 mice per group. Statistical significance was determined by Kruskall Wallis test. * *P* < 0.05 and ** *P* < 0.01.


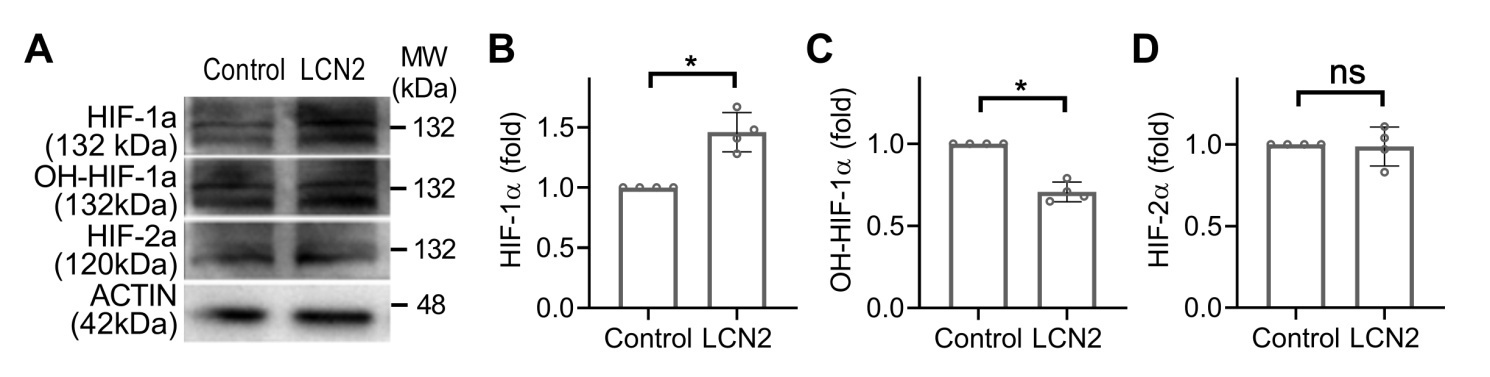


**Figure S7. LCN2 induced HIF-1α expression in PASMCs.** In vitro cultured human PASMCs were treated with LCN2 (2 nM) or saline for 24 hour. Representative Western blot images **(A)** and quantification analyses **(B-D)** of the protein levels of HIF-1α, Hydroxylated HIF-1α (OH-HIF-1α) and HIF-2α were shown (n=4, independent experiments). Results are expressed as mean ± SE. Statistical significance was determined by Mann Whitney U test. * *P* < 0.05 and ns *P* > 0.05.
